# Involving Service Users in Care Regulation: A Scoping Review of Empirical Literature

**DOI:** 10.34172/ijhpm.8509

**Published:** 2025-03-18

**Authors:** Josje Kok, Flora Palimetaki, Nada Akrouh, Linda Schoonmade, Hester van de Bovenkamp, Anne Margriet Pot

**Affiliations:** ^1^Erasmus School of Health Policy & Management, Erasmus University Rotterdam, Rotterdam, The Netherlands.; ^2^Dutch Health and Youth Care Inspectorate, Utrecht, The Netherlands.; ^3^University Library Vrije Universiteit, Amsterdam, The Netherlands.; ^4^North-West University, Vanderbijlpark, South-Africa.

**Keywords:** Scoping Review, Regulation, Supervision, Quality and Safety, Service User Involvement, Participatory Practices

## Abstract

**Background:**

Ensuring the quality and safety of service delivery extends beyond the realm of health and care professionals, necessitating collaboration among various stakeholders, including external regulatory organizations. The policy agenda of care regulators increasingly features the topic of service user involvement. Despite the extensive research on participatory healthcare, scholarly attention to service user involvement in regulatory practices has been limited. This scoping review delves into the landscape of service user involvement in the regulation of care services of all types and for all different age groups, examining the characteristics and focus of peer reviewed original research. In particular, it addresses a notable knowledge gap by examining how these studies report on the practical utilization of service user input, as well as the regulator’s perspective on service user involvement.

**Methods:**

We conducted a literature search in PubMed, Embase, CINAHL, APA PsycInfo, and Scopus from inception to July 14, 2023. Thirteen (n=13) empirical studies were included.

**Results:**

The underlying motives for service user involvement vary, ranging from legal imperatives and political pressure to enhancing institutional legitimacy and regulatory decision-making. Care regulators employ both reactive and proactive involvement methods. Empirical evidence delineates the challenges and benefits of service user involvement, highlighting concerns about bias, time investments, and the need for a distinct skillset for inspectors. Despite the valuable insights gained, there are instances where service user input is downplayed in practice.

**Conclusion:**

The findings underscore the importance of additional research on users’ preferences for involvement, optimal communication conditions to honor the collected input, and the challenges inspectors encounter in fostering meaningful involvement with service users. Addressing these challenges is crucial for aligning regulatory efforts with the genuine needs and experiences of services users.

## Background

 “*We want to be an advocate for change, with our regulation driven by people’s needs and their experiences of health and care services, rather than how providers want to deliver them. This means focusing on what matters to the public, and to local communities, when they access, use and move between services. Working in partnership with people who use services, we have an opportunity to help build care around the person: we want to regulate to make that happen*^[1]^.*”*^1^

 In many health and care systems, national regulatory bodies are tasked with overseeing and enhancing the quality and safety of service delivery.^2-4^ That is: safeguarding the quality and safety of care is not solely the responsibility of healthcare professionals; it involves various stakeholders, including regulators.^5,6^ While definitions of regulation vary based on professional discipline, political ideology, and even geography,^7,8^ it generally refers to the oversight and enforcement of rules, standards, and policies that ensure the quality, and safety of services and products. The role of care regulators varies by country, depending on national laws and health- and care systems. Their work often includes the setting and/or enforcing of standards, conducting inspections, monitoring performance and outcomes, and ensuring compliance with regulatory requirements. In this paper, we focus specifically on the work of national care regulators, appointed by the government to oversee the delivery of high-quality and safe care.

 Within regulatory regimes the topic of service user involvement has steadily gained more prominence on the policy agenda.^9,10^ In the context of health and social care, any individual may, at some point, become a service user or have caregivers who rely on these services, creating a vast potential for valuable input. Consequently, care regulators across the globe—spanning from England to the United States, and from the Netherlands to Australia—increasingly view patients, clients, and their representatives as knowledgeable stakeholders, and even as partners, as depicted in the opening quote.^1^ More and more, the insights and input from (potential) service users and their relatives are considered crucial in guiding regulators in their oversight activities. This includes deciding the focal points of regulation, determining what “good” quality and safety entails, and assessing quality in practice.^1,11,12^

 The premise that it is insufficient for a regulator to act on behalf of people needing and receiving care, without actively involving them in its work stems from several socio-political developments.^10,12^ The first socio-political development refers to a broader trend in public policy and governance, where the involvement of citizens in decision-making is emphasized. This development has been driven by the principle that individuals affected by decisions should be granted a chance to actively engage in the corresponding decision-making processes.^13^ Second, we currently live in an age where public trust in institutions is no longer a given.^14^ Regulatory organizations thus face mounting pressure to engage the public in decision-making processes and policy development in order to establish and maintain legitimacy and societal value.^10,15-19^ Thirdly, specifically within the context of health and care services, the stronger emphasis on person-centeredness—including processes of shared-decision-making—have elevated it as a key quality requirement for service delivery. This shift has pushed professionals, service providers *and* their regulators to actively seek-out and understand what it is that service users need and value and move beyond mere quantifiable and objectifying metrics.^5,20^ As such, the person-centeredness movement has prompted regulatory organizations to find ways to monitor and assess if providers indeed prioritize and attend to the needs and preferences of their users, both in routine service delivery and in situations where issues arise or things have gone wrong.^18,21^

 While there exists a substantial body of literature on the involvement of users in service delivery, patient safety and quality improvement (See for instance^17,22-24^), less scholarly attention has been awarded to the involvement of patients, clients, and their representatives in regulatory practices.^16^ A cross-country overview demonstrated four ways of involving service users in healthcare regulation, namely: individual-proactive, collective-proactive, individual-reactive and collective-reactive approaches (See Table 1 for details and concrete examples).^9^ Building on these insights, a recent review study concentrated on categorizing these approaches into low, medium and high forms of participation.^25^ This classification does not, however, provide a clear understanding of why regulators involve users and how user input is actually utilized. In-depth insights into these aspects can be obtained by critically examining existing peer reviewed original research, as done in this paper. Furthermore, we expand on the aforementioned reviews by specifically highlighting the regulator’s perspective – focusing on how inspectors perceive and experience service user involvement.

**Table 1:** Different Methods Used to Promote Service User Involvement in Healthcare Regulation^a^

**Individual-Proactive**	**Collective-Proactive**
Refer to involvement of individuals with the purpose of collaboration and use of information for setting the future regulatory agenda and planning regulatory activities *Examples* Interviews with clients during planned inspections (as done in England, the Netherlands, Norway, among others) Annual service user surveys to collect experiences (as done in England and Australia)	Approaches that involve users/‘experts,’ who are expected to represent a group of interests with the purpose of informing the future regulatory agenda or specific regulatory activities, such as inspection visits *Examples* Use of experts-by-experience & mystery-guests during inspections (as done in Australia, England, the Netherlands, Norway, among others) Using client experiences as a source of information for reports and future theme-based inspections (as done in England, the Netherlands and Norway)
**Individual-Reactive**	**Collective-Reactive**
Refer to methods regulators use to involve individuals (patient, client, next-of-kin) when they have experienced either an adverse event or filed a complaint about the service provision to the regulator *Examples* Attending to service user complaints (as done in England, the Netherlands, Norway, Australia, among others)	Collective involvement after service provision failures or complaints. The involvement is not focused on an individual’s own case or experience *Examples* Aggregated information from complaints on sector and themes as used in risk-based supervision for agenda setting (as done in England and the Netherlands)

^a^Taken from Wiig et al,^9^ p. 6-7.

 In this scoping review we focus on peer reviewed original research, concerning the involvement of service users in care regulation and map the characteristics and range of existing studies, with the aim to foster person-centered service delivery and quality improvement, as well as identify knowledge gaps for further research. Notably, when we use the term “care” we refer to services for people of all ages and needs across health and social care settings, including hospitals, general practitioners, and long-term care (LTC) environments such as home care and residential facilities. As scoping reviews are suitable for looking at emerging innovative fields and developments,^26^ our research questions were exploratory, broadly interested in depicting (*a*) why and how regulatory organizations collect and/or use service user input, (*b*) how regulators experience these involvement practices, and (*c*) how the input provided by service users is utilized.

## Methods

 The description of this scoping review aligns with the Preferred Reporting Items for Systematic Reviews and Meta-Analysis extension for Scoping Reviews (PRISMA-ScR) statement.^27^

###  Search Strategy

 A comprehensive search was performed in the bibliographic databases PubMed, Embase.com, CINAHL (via Ebsco), APA PsycInfo (via Ebsco) and Scopus from inception to July 14, 2023, in collaboration with a medical librarian (LS). Search terms included controlled terms as well as free text terms. Search terms expressing “inspection” and “regulation” were combined with search terms comprising (variants for) “client experience” and (variants for) “healthcare facilities,” “long-term care” and “social care.” The search was performed without date or language restrictions. Duplicate articles were excluded by a medical librarian (LS) using Endnote X20.4 (Clarivate^TM^), following the Amsterdam Efficient Deduplication (AED)method and the Bramer-method.^28^ The full search strategies for all databases can be found in Supplementary file 1. The review protocol has not been registered.

 To complement the database search, the first author (JK) performed a backward snowballing hand-search using the articles that were selected (See Figure).^29^

**Figure:**
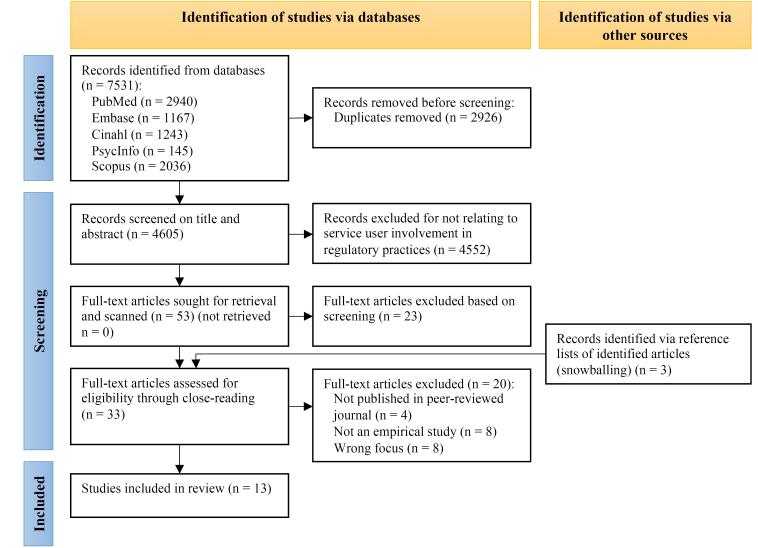
Flowchart of the Search and Selection Procedure of Studies. Five databases were searched from data of inception to July 2023. See Methods sub-section ‘Selection Process’ for inclusion and exclusion criteria.

###  Selection Process

 The selection process consisted of two screening rounds, with meetings after screening. First, authors FP and NA, independently screened all records on potentially relevant studies based on titles and abstracts, using Rayyan.^30^ Articles were included if they met the following criteria: (1) empirical studies (qualitative and/or quantitative); (2) focus on regulatory organization for health and/or care services (including hospitals, LTC facilities, youth-care, etc); (3) that collects and/or uses client, patient, relatives and/or lay-person’ experiences and/or views, and; (4) uses these in one or more of its regulatory activities (eg, inspection visits and audits, institutional risk-assessments, providing advice, taking disciplinary measures, etc). Studies that met these inclusion criteria but were (1) published in another language than English, and (2) not published in a peer-reviewed journal were excluded. Consequently, all gray literature was omitted. In the second screening round full-text articles were divided and FA and NA conducted closer readings to assess eligibility. HvdB assisted in this round by evaluating articles that prompted uncertainty regarding eligibility. In a last meeting with authors AM and HvdB uncertainties were discussed, and consensus was reached on the final selection (n = 13, see Table 2). Given our specific interest in the way in which regulatory organizations collect and/or use service user input in their regulatory routines, we excluded empirical studies that for example: (1) report on ways in which regulatory organizations promote and oversee the involvement of clients, patients, and their next-of-kin on the part of service providers (eg,^31^) or in cases of service failures, such as adverse event investigations^32-35^; (2) discuss correlations between (online) service user ratings and regulatory quality assessments of LTC facilities without these online ratings actually being part of regulatory activities (eg,^36^); and (3) merely mention that patients/clients play a role in regulatory activities without providing any further details and insights on the way that this is actually done in practice (eg,^37^).

**Table 2:** Studies Included in the Review

**Grouping Category**	**Reference: First Author & Year**	**Country & (Social) Care Setting**	**Study Design & Method(s)**	**Study Objective**	**Type of User Involvement & (If Described) How Input Is Used**	**Underlying Rationale/Motive for Involvement**
Strategy 1: Service user input used for risk-assessments	Bouwman et al,^39^ 2015	The Netherlands; Hospitals, LTC-facilities general practitioners, pharmacies, etc.	Mixed-methods: Survey and qualitative interviews.	Assess expectations and experiences of service users who complained to the regulator.	Individual-reactive or collective-reactive (depending on nature of the complaint): Citizens can report complaints to the Inspectorate via telephone or through a digital platform. Complaints relating to severe or structural issues are handled by the Inspectorate. Inspectors determine the seriousness/severity. How this is done is not specifically described.	Legal requirement for Inspectorate to offer a platform to report complaints. Attending to complains is seen to be important for regulator’s institutional-legitimacy.
	Griffiths et al,^40^ 2018	England; Hospitals/Trusts.	Quantitative: Logistic regression analysis of the relationship between a collective judgement score on the start date of hospital and Trust-level inspections, and the subsequent inspection outcomes.	Determine whether automated collection and aggregation of multiple sources of online patient feedback can provide a collective judgement that effectively identifies risks to the quality of care, and hence can be used to help prioritize inspections.	Individual-proactive & collective-reactive: Patient-feedback and ratings from rating and social media websites transferred into CJS. CJS’s were then paired with the overall rating from scores from earlier CQC inspection visits.	Providing a different perspective on quality to traditional metrics. Online user input timelier (near real time) and at a more granular (hospital) level, than the data typically used by regulator.
	Van de Belt et al,^41^ 2015	The Netherlands; LTC- facilities, hospitals and home care services.	Qualitative: Review and categorizing of quantitative and qualitative online voiced care-related experiences; added value of this information rated by inspectors.	Identify the added value of healthcare-related experiences and ratings voiced on social media for the regulator’s risk-based and incident-based supervision programs.	Individual-reactive (for incident-based regulation) or collective-reactive (for risk-based regulation): User feedback on social media and rating site searched for specific risk-based themes and incidents; mapped to determine if searches provided information that was of additional value for the regulator.	Additional source of information, potentially valuable to regulators to help prioritize. Information from service users seen as potentially more timely/current (in comparison to, for instance, yearly inspections).
Strategy 2: Service user input used to assess quality of care	Braithwaite et al,^42^ 1992	Australia; LTC-facilities for older persons.	Quantitative: Data from two inspections of a random sample of nursing homes with a 18-20 month follow-up period, compared to aggregated national data on residents’ socio-economic profile, care needs and behavioral problems.	Determine whether a resident-centered inspection process can be effective in a nursing home environment dominated by residents who require high levels of care.	Individual-proactive: Residents are interviewed by inspectors during inspection visits, and asked about their opinion on topics related to the outcome standards inspectors assess. Subjective experiences of residents used to judge if standards are met.	Providing a different perspective on quality to traditional measurable quality metrics. Clients seen as valuable “knowers” of the care they receive.
	Rutz et al,^43^ 2018	The Netherlands; Health and social services that provide help to children growing up in poverty.	Qualitative: Document analysis and focus groups.	Analyze how inspectors from the Joint Inspectorate Social Domain involved the perspectives of young care users in a theme-based inspection program, and assess how inspectors deal with similarities and differences to their own views.	Collective-proactive: Focus-groups and interviews with adolescents as part of theme-based inspection process. Children were invited to share their experiences, through interviews and focus-group discussions during inspection process. Topics included: what young people considered poverty, how they experienced their situation, whether they had received care and assistance, how they experienced this and what they considered to be necessary improvements for young people living in poverty.	Clients seen as valuable knowers/having a different perspective. Involvement seen as a way to strengthen regulatory decision-making.
	Pålsson,^44^ 2017	Sweden; Residential care homes for children.	Qualitative: Document analysis; observations and interviews.	Describe and analyze what influence the inspectorate grants children in care during the inspection process and particularly how children’s views influence the inspection process.	Individual-proactive: Residents (children) interviewed during inspection visits about their experiences with the inspected regulatory norms. Findings can be taken up in inspection report.	Legal requirement that children are consulted during inspection visits.
	Verver et al,^45^ 2018	The Netherlands; LTC-facilities for older adults living independently.	Qualitative: Semi-structured interviews and focus groups.	Study both the added value to and the barriers experienced with the use of a newly developed regulatory framework focusing on care-networks.	Individual-proactive: Clients and their informal carers recruited to be interviewed by inspectors, interviews structured along the lines of predefined themes in the regulatory framework. Input provided by users used to map the network of service providers and assess the functioning of this network according to the clients’ perspective.	Changing healthcare landscape forcing regulator to rethink its traditional regulatory approach. Clients seen as valuable knowers on the care network they use.
Strategy 3: (Potential) service users used as collectors of information	Adams et al,^46^ 2015	The Netherlands; LTC-facilities for older persons.	Qualitative: Interviews, observations, document and web analysis.	Determine if and how “mystery guests” can be used as an instrument in the supervision and regulation and what types of information or insights about daily care the “mystery guests”’ could deliver.	Collective-proactive: Unannounced inspection visits by “mystery-guests,” who reported on their institutional reviews (“portraits”) on the mystery-shopper website.	Project initiated after political pressure. Involvement seen as way to incorporate additional information into regulatory process. Enhance trust in the regulator /improve institutional legitimacy.
	de Graaff et al,^15^ 2019	The Netherlands; LTC-facilities for older persons.	Qualitative: Observations, document analysis and interviews.	Describe the use of experts-by-experience to evaluate the quality and safety of nursing homes and discuss how, and what kind(s) of knowledge is produced and legitimated using experts-by-experience as an instrument of supervision.	Collective-proactive: Experts-by-experience joining inspectors during inspection visits. Experts-by-experience joined inspection team during annual institutional review site visits. They conducted semi-structured interviews with clients and were asked to make/note down observations on template forms.	Regulator under societal & political pressure to involve service users. Involvement seen as way to legitimize decision-making processes. Enhance trust in the regulator /improve institutional legitimacy.
Combination of strategies 1, 2 & 3	Richardson et al,^18^ 2019	England; Health and social care in its entirety, incl. hospitals LTC-facilities, adult social care and general practitioners.	Qualitative: Document analysis and interviews.	Explore the role of service users and citizens in health and social care regulation, including how CQC involved people in inspecting and rating health and social care providers.	Individual-proactive, collective-proactive and collective-reactive: Different types of involvement: gathering general information such as complaints and compliments from users prior to an inspection, as well as assessing routinely collected user feedback via national and local surveys; using experts-by-experience during inspections; speaking to local service user groups to inform a forthcoming inspection.	Involvement strategies amplified/introduced after social and political pressure.
Expectations and experience of inspectors using clients’ perspectives	Adams et al,^16^ 2013	The Netherlands; Healthcare in its entirety.	Qualitative: Document and web analysis, focus groups and interviews.	Determine what the (political) expectations for increased citizen participation are, and how these expectations compare to regulators’ expectations and experiences in practice.	Depending on strategy, individual-proactive, individual-reactive, collective-reactive and collective-proactive Different types of involvement: providing information to service users (and managing their expectations); including citizens as information sources via interviews during inspection visits, web-based surveys and using complains and incident reports; stimulating and monitoring how regulates involve their service users to work on quality and safety. Information provided by service users used by inspectors to sensitize them to issues or important themes that they would have missed via indicators or standardized inspection visits.	Involvement seen as way to legitimize decision-making processes. Enhance trust in the regulator/improve institutional legitimacy.
	Wiig et al,^47^ 2019	Norway; Healthcare in its entirety, incl. hospitals, primary care, emergency services, etc.	Qualitative: Observations and focus group interviews.	Explore regulatory inspectors’ experiences with a new method for next-of-kin involvement in investigation of fatal adverse events.	Individual-reactive: Next-of-kin interviews by inspectors: family members who experienced the loss of a family member in an adverse event, consulted in 2 hour face-to-face meeting. Interviewed about their experiences as input for incident investigation.	Project initiated after societal and political pressure. Next-of-kin seen has having a different/additional perspective.
	Kleefstra et al,^48^ 2016	The Netherlands; Hospitals.	Mixed-methods: Interviews, review and scoring relevance of online ratings.	Explore whether and how patient experiences reported on rating sites can, in the eyes of inspectors, contribute to risk identification in hospital care.	Collective-reactive or individual-reactive: Online service user-feedback and ratings. Inspectors consult online rating site to prepare for their annual meeting with hospital boards or in case of reports of serious incidents. Some inspectors scan social media channels (eg, Twitter) and news sites to gather information.	Input seen as an additional source of information.

Abbreviations: CQC, Care Quality Commission; CJS, collective-judgement score; LTC, long-term care.

###  Data Charting Process

 After the selection phase, data was systematically charted and synthesized.^38^ Authors JK and FP mapped details of included articles in a standardized spreadsheet, including study author(s), year, country, care setting, study design, and objective(s) as well as a description of the type of service user involvement.

###  Synthesis of the Results

 Using this spreadsheet, the first author (JK) grouped the studies according to overarching themes, see Table 2. In several joint meetings, the authors discussed and refined the grouping categories. Finally, the second author (FP) conducted a last thorough review of the spreadsheet, double-checking the grouping, characteristics, and findings to ensure accuracy and consistency.

## Results

 Figure outlines the identification of studies through databases and snowballing.^49^ Additionally, it delineates the number of records screened and subsequently excluded. Ultimately, 13 studies were included.

 The remainder of the results section is structured around our exploratory research questions. We begin with an overview of the empirical studies, offering a high-level summary of their characteristics, focus areas, and the rationales behind service user involvement. Next, we examine the methods of involvement outlined in the studies, detailing how care regulators implement these methods within various strategies, which are discussed sequentially. Finally, we explore inspectors’ expectations and experiences regarding service user involvement.

###  Study Characteristics

 All 13 studies have been carried out in high-income countries: Australia (n = 1) England (n = 2), the Netherlands (n = 8), Norway (n = 1), and Sweden (n = 1) (See Table 2, column “Country & (Social) Care Setting” for all details). The oldest study dates to 1992 (Australia), while all others were conducted between 2013 and 2019. The studies report on service user input that was collected and/or used for regulatory practices monitoring risks or targeting the quality and safety of hospitals and Trusts (n = 2), LTC for older persons in LTC-facilities (n = 3) and LTC for older persons in home settings (n = 1), youth care facilities (n = 2), or a combination of different health and care services (n = 5). None of the identified studies specifically discuss service user input collected for the regulation of hospital at home services, or other types of acute home-care settings as monitored by national regulators. The research methods used are qualitative (n = 9), quantitative (n = 2) or mixed-methods (n = 2).

###  Focus of Studies

 The empirical research delved into different aspects of service user involvement in regulatory practices and were categorized accordingly. Specifically, ten articles scrutinized service user involvement as a deliberate regulatory strategy. Our analysis unveiled three strategies: (1) service user input used for risk-assessments (n = 3); (2) service user input used to assess quality of care (n = 4), and (3) (potential) service users used as collectors of information (n = 2). In daily regulatory practice, it is important to acknowledge that these three strategies can coexist. Notably, service user input addressing a quality issue may also point to persistent risks that warrant attention, and vice versa.^20^ For the purpose of this review, we have grouped these strategies separately based on the regulators’ initial plan for the utilization of service user input. In addition to these three strategic categories, one study from England discussed a combination of these three strategies (n = 1). Lastly, three studies specifically scrutinized the expectations and experiences of inspectors regarding service user involvement (n = 3).

 The analysis also yielded insights on the different underlying rationales for user involvement in regulatory practices and the varied methods employed by care regulators. In what follows we first detail these rationales and methods, and then delve into the grouped categories discussing the studies in more detail.

###  Underlying Rationales for User Involvement

 Regulators are often driven by multiple underlying motives (See Table 2). A first motive for service user involvement is that it is a legal requirement, forcing regulators to organize these practices.^39,44^ Moreover, some studies indicate that the implementation of service user involvement practices was prompted by political and social pressures.^15,18,45,47^ In connection with these political and societal pressures, maintaining a regulator’s institutional legitimacy, building public trust, and enhancing regulatory decision-making are also seen as important drivers for organizing service user involvement.^15,16,39,43,46^ Lastly, service users are perceived to potentially contribute additional information, serving as an *extra set of eyes* that can help regulators prioritize and provide insights in a timely matter,^41,46-48^ or as *a different set of eyes* that can furnish new insights, potentially complementing or diverging from the perspective of regulators.^40,42,43,45,47^

###  Involvement Methods

 The empirical studies examine various proactive and reactive service user involvement methods employed by care regulators that can be categorized using Wiig and colleagues’ typology (Table 1).^9^ Individual-proactive methods include local surveys, regulator-issued questionnaires, and client interviews during inspections.^16,18,40^ Collective-proactive involve client interviews,^45^ using experts-by-experience,^15,18^ and deploying mystery guests.^46^ Regulators predominantly use these proactive methods to gather information on care quality within specific regions or providers. The studies also highlight individual-reactive methods, such as complaints handling,^18,47^ scanning feedback on social media,^40,41,48^ and conducting interviews with next-of-kin in serious incident investigations.^47^ Collective-reactive methods involve leveraging aggregated information from complaints,^18,39^ and monitoring online user feedback to set the regulatory agenda.^41^ All reactive methods, except for addressing individually issued quality complaints and involving next-of-kin in incident investigations, are employed by regulators to monitor and determine (structural) risks to quality and safety. These exceptions may also be utilized to address urgent quality concerns that require immediate attention.

###  Strategy 1: Service User Input Used for Risk-Assessments

 Three studies focus on the utilization of service user input for risk-assessment purposes, through the monitoring of complaints and processing of voiced feedback on social media and websites. With regards to service user complaints, Bouwman et al^39^ foregrounds how the Dutch Health and Youth Care Inspectorate (HYCI) processes service user complaints. The HYCI is tasked with addressing complaints from service users only in cases of severity (eg, deliberate misconduct, sexual abuse, etc) or when they indicate underlying structural problems, suggesting ongoing risks. The study shows that complainants are aware that the HYCI does not attend to individual grievances. At the same time there is a discrepancy in complainants’ perceptions of the relevance of their complaints (ie, pointing to deeper structural problems that may reoccur) compared to inspector assessments. Furthermore, a mere minority of individuals whose complaints were handled by the HYCI, felt that their grievances resulted in quality and safety improvements, which was their primary reason for issuing a complaint.

 Belt et al and Griffiths and Leaver, explore the added value of information on social media for the regulator’s supervision of healthcare services.^40,41^ Belt et al, concludes that information from a Dutch service user rating site offers valuable information for the HYCI’s risk-based regulatory program, leading inspector’s to relevant (new) signals that warrant further research. The HYCI has incorporated the ratings from the rating site into their risk-based surveillance system.^41^ Other online sources, such as Twitter and Facebook, did not yield valuable information for regulatory purposes, mostly because posts were not detailed enough.^41^ In Griffiths and Leaver’s study^40^ Twitter and Facebook posts *are* used and combined with unsolicited feedback for all NHS (National Health Service) Trusts and hospitals from a government-run website. They conclude that this input, combined into a collective judgement score, can effectively identify high-risk groups of organizations, and hence can be used to prioritize CQC’s inspections.

###  Strategy 2: Service User Input Used to Assess Quality of Care

 Four studies specifically focus on the use of service user input as a formal part of regulatory practice to assess the quality of service delivery. This is done through client interviews during inspection visits as well as service user focus-groups. In each of these studies, user input is consistently emphasized as crucial for making quality assessments. Effectively incorporating user input into regulatory assessments poses challenges. It requires specific skills from inspectors and in practice doing justice to user input appears to be a complex task.

 The oldest study in this review, dating back to 1993, by Braithwaite et al details how residents in Australian LTC-facilities for older persons are interviewed by inspectors and concludes that residents are an important source of information to determine compliance to a variety of “quality of life” outcome standards developed by authorities. Importantly, the authors argue, these client-interviews require “highly skilled inspectors,” who play a key role in obtaining useful information. Skilled inspectors excel in identifying approachable interviewees; adeptly detect cues; employ creative ways to communicate with residents; and triangulate input with other sources of information such as documentation, management interviews and observations.^42^

 Beyond the importance of an inspector’s skillset, three studies elaborate on the significance of the “regulatory rationale” in shaping the assessment of service user input as “useful.” A Swedish study by Pålsson describes how children in residential care are consulted by inspectors when these services are reviewed.^44^ The study demonstrates difficulties in giving children’s views substantial impact on the inspection process. This is ascribed to the divergence between the regulatory quality criteria employed by the inspectorate and the aspects of care that hold importance for children. Pålsson terms this misalignment as a “regulatory rationale,” wherein client opinions are considered pertinent only to the extent that they furnish tangible evidence of an institution’s compliance or non-compliance with regulatory requirements.^44^

 Dutch studies by Rutz et al^43^ and Verver et al^45^ also demonstrate the dominance of the “regulatory rationale.” Rutz et al delve into how inspectors from the Joint Inspectorate Social Domain involved the perspectives of young care users in an inspection-process focused on a broad range of social and healthcare services dedicated to assisting children growing up in impoverished conditions.^43^ Data showed that although inspectors acknowledged what adolescents thought, they often awarded their own view more weight. Inspectors did incorporate adolescents’ views in the formal inspection reports; they used adolescents’ information to substantiate and illustrate their view (when the perspectives were similar) and they documented the information separately from inspectors’ views (when their views differed). Rutz et al found no examples of inspectors changing their opinions based on the views of adolescents.^43^ Verver et al investigated the added value of a newly developed (experimental) regulatory framework by the HYCI, focusing on care-networks for people receiving LTC at home.^45^ Inspectors interviewed individual clients and their informal carers to map their network of service providers used and, in turn, assess the functioning of this network according to the client’s perspective. Like Pålsson^44^ and Rutz et al^43^ the results reveal however that what was important for clients (eg, kindness of professionals and making time to have a conversation) did not always coincide with what inspectors and professionals considered to be important aspects of quality and safety. Here too the norms in the inspection framework were not changed based on the perspective of clients. The authors thus conclude that despite—the costly and time consuming—efforts made to directly involve clients as a source of intelligence, the ‘professional perspective’ still prevailed.

###  Strategy 3: (Potential) Service Users Used as Collectors of Information

 Two studies from the Netherlands report on pilot projects conducted by the Dutch HYCI, in which (potential) service users were utilized as collectors of information, in the hope that they would provide a new and unique perspective to the regulatory-assessment process.^15,46^ In practice, both studies demonstrate that it was difficult for inspectors to use and embrace a “unique” perspective. In the first project, “mystery-guests” were implemented as an instrument of supervision in long-term intramural care facilities for older persons.^46^ “Mystery-guests” are undercover evaluators, akin to mystery-shoppers commonly employed in commercial retail settings. They carry out surprise visits to evaluate and provide feedback on service delivery. The expectation that mystery-guests would provide a better view of the exigencies of daily care practice was not met. Inspectors did not trust and use the information delivered by the mystery-guests because of how they evaluated quality (focus on “soft aspects” of care) and reported their findings (mostly narratively and not always supported by “verifiable” evidence) did not align with the HYCI’s common practices.

 In a second project, experts-by-experience were trained by the HYCI to join inspectors on LTC-facilities inspection visits.^15^ The authors conclude that for the Inspectorate it was labor intensive to work with experts-by-experience, yielding—in line with the regulator’s standards—limited additional insights on the quality of service delivery. Experts-by-experience conducted interviews with clients, made observations, and reported their findings to inspectors, who incorporated them into their data for formal assessments. However, information deviating from inspectors’ findings, such as notes on clients’ daily lives, was deemed less valuable, as inspectors struggled to verify these details. So whilst the experiment intended to open-up valid new experiential knowledge of clients to improve the regulation practices, the chosen methodology of the pilot (using formal training practices, etc) structured the practices in such a way that this “different” knowledge was unlikely to be opened-up.^15^

###  Combination of Strategies 1, 2, and 3

 Richardson et al conducted an inductive analysis of data collected three years after the strategic overhaul of the CQC’s regulatory regime.^18^ The study explores the role of service users and citizens in CQC’s regulation, encompassing activities from gathering service user feedback through local surveys (for risk-assessment purposes) to service user interviews (for quality of care assessments) and the use of experts-by-experience on inspection visits (service users gathering information). Challenges in involving service users include difficulties in being responsive to individual concerns while maintaining professional distance and objectivity. The encounters between CQC and service users are deemed somewhat transactional, serving CQC functions and processes without building enduring relationships with local service user groups. Also, despite good intentions, the authors report there was a lack of transparency in how service user input was incorporated into the inspection and rating process, and involvement often ends after sharing experiences with CQC.

###  Expectations and Experiences of Inspectors With Service User Involvement

 Three studies specifically scrutinize the expectations and experiences of inspectors with different service user involvement approaches. The studies demonstrate that inspectors acknowledge the significance of involving service users in regulatory practices, yet express concerns about the validity of user input. Furthermore, they perceive it as time-consuming and argue it necessitates a (new) skillset.

 In the Norwegian study, Wiig et al describe a new method for next-of-kin involvement in the investigation of fatal adverse events.^47^ The inspectors experienced next-of-kin involvement as a method that informed and improved the quality of the investigations by adding new and important information. At the same time, meetings were experienced as emotionally challenging and increased inspectors’ workloads significantly. Lastly, inspectors missed sufficient resources (time and funding) to follow up on new information that stakeholders identified in the meetings.

 A Dutch study by Kleefstra et al, investigates the potential contribution of patient rating sites to hospital supervision practices.^48^ The study demonstrates how inspectors consult an online rating site to prepare for their annual meetings with hospitals boards, or in case of serious incidents. Some inspectors scan social media channels such as Twitter and Facebook, to gather background or contextual information. Although inspectors do consult these online platforms to incorporate service user experiences as an additional source of intelligence, inspectors do have reservations about the usefulness of this information. They worry about the subjectivity and representativeness of the information. Concerns about representativeness also came to the fore in the study by Adams et al.^16^ What’s more, Adams et al reveal, like Braithwaite et al,^42^ that involving service users demands specific skills, that not all inspectors have. Inspectors experienced service user involvement as time-consuming and at times difficult to organize and call for carefully considering what type of involvement is relevant in a specific situation.

## Discussion

 In this scoping review we have mapped the characteristics, range and focus of empirical evidence pertaining the involvement of service users in the regulation of care. The review identified 13 empirical studies, with a singular exception,^42^ all conducted within the last decade. The studies focus on different aspects of service user involvement: shedding light on various underlying rationales, strategies, and ensuing methods, as well as inspector experiences. All studies were from high-income countries, including Australia, England, Norway, Sweden, and the Netherlands in particular (n = 8).

 Regulators involve service users in their regulatory practices in different ways, using reactive as well as proactive methods. Nine out of 13 studies describe methods that have been newly introduced or have a pilot-character. With one exception,^45^ the proactive methods studied focus on collecting quality information for individual providers rather than – for example – networks of providers. Proactive service user involvement tends to be dictated by the regulator’s terms, where, for instance, experts-by-experience address topics predetermined by the regulator, and clients are interviewed based on predefined topic lists or structured questionnaires.

 Our analysis builds on two earlier reviews on service user involvement,^9,25^ by specifically foregrounding the empirical findings related to the regulator’s perspective on this involvement. Our study demonstrated that inspectors find service user involvement valuable, pointing to instances where users offered new insights otherwise overlooked, as is once again confirmed by a recent Dutch pilot-study on individuals with intellectual disabilities serving as mystery guests.^50^ Nevertheless, many inspectors also have concerns about bias and struggle with the verifiability of the input that is collected. Involvement practices are time consuming and some studies point out inspectors lacked the support and resources to follow up on input properly.^16,18,47^ Lastly, inspectors emphasize that involving service users, especially when direct engagement, in the form of face-to-face contact, is necessary, demand a distinct skillset and different role, which not all inspectors possess (yet).

 Regulatory organizations have diverse and often simultaneous motives for involving service users, including legal reasons, political and social pressure, and the wish to increase their legitimacy and quality of regulatory oversight and decision-making (See Table 2, column “Underlying Rationale/Motive for Involvement”). In the analysis we have relied on the motives documented by the researchers/authors, in practice more or different rationales may exist (See the discussion of the limitations of this study, below). Nevertheless, the analysis does give a general overview of the various rationales influencing (the commencement of) service user involvement practices. Notably, the studies did not assess whether the introduced involvement approaches effectively advanced legitimacy, trust, and improved decision-making from the perspective of service users. This is an important avenue for further research.

 The central underlying motive to involve service users is based on the premise that they can offer valuable input. The relevance of this input if framed in two distinct ways. Firstly, users are perceived as potential sources of additional information, for instance by providing new—earlier overlooked—details in an adverse event investigation,^9^ or by (reactively or proactively) providing feedback online on services being monitored by a regulator.^18,48^ Secondly, some studies show that service users are seen as being valuable knowers in their own right, holding a different perspective that can complement that of the regulator.^43-45^ In presenting a *different* perspective, however, the studies unveil that service user input is often downplayed if their views diverge on the concept of quality or what is significant for them.^15,46^ Additionally, when viewpoints conflict, the perspectives of service users do not appear to influence the judgements made by inspectors; inspectors’ assessments of quality and safety risks carry more weight.^15,43-46^

 Just recently, the use of experts-by-experience and utilization of mystery guests have been labeled as “higher-level” participation forms,^25^ referring to methods based on deliberative communication between users and care regulators. “Medium-level” participation refers to methods that are based on two-way communication forms, such as user interviews and focus-groups. However, regardless of the categorized level, this scoping review demonstrates that even with deliberative communication practices and dialogue, service users risk their perspectives to be downplayed if regulators do not reflect on their dominant interpretative and assessment frameworks. This observation is significant, as it underscores the importance of crafting and evaluating participatory methods in alignment with their underlying strategies and motives. Moreover, this observation draws attention to the idealized view of regulatory practices as entirely objective and standardized.^51^ The dismissal or undervaluation of information provided by service users is not merely a consequence of individual inspectors’ skillsets or resource constraints. As previous research has shown, it can also stem from institutionalized logic about which representations and perspectives are considered valid-or less important. Addressing this issue requires organizational-level changes and support that foster (interorganizational) dialogue and reflection on how inspectors integrate and weigh different types of information in their decision-making processes.^35^ If the ambition is to offer users a “seat at the table” to learn from their experiences and unique perspectives to help build care around them,^1^ users must be provided with the room to voice those perspectives and experiences in a manner that does justice to their epistemic contribution.^35,52^ Importantly though, embracing a “different perspective” requires regulatory organizations to be reflexive of their own interpretative and regulatory frameworks.

 Considering these findings there is a need for further research on participatory practices within care regulation, delving into users’ preferences for involvement, the conditions necessary for effective communication, and the potential challenges faced by inspectors in facilitating such direct involvement. Moreover, it is essential to examine the skillset inspectors must need and organizational (strategical) backing to effectively work with service user experiences.

 This review has several limitations. First, by focusing solely on peer-reviewed publications, we have overlooked insights from gray literature, such as internal reviews and evaluations conducted by regulatory organizations. These omissions could have created blind spots in our analysis. Given the growing interest in this topic from both regulators and regulatory scholars, we recommend conducting a follow-up scoping review in a few years to capture emerging methods, utilization strategies, and experiences. Additionally, there may be a geographic bias in our findings, as many of the empirical studies originate from the Netherlands. This overrepresentation is likely due to the Dutch HYCI’s longstanding academic collaborative center on healthcare regulation, which has produced significant research on its regulatory practices, including service user involvement.^53^ Incorporating gray literature from other countries in future reviews could help address this imbalance and provide a more comprehensive understanding of international perspectives and challenges. To build on the insights we have gained about inspectors’ experiences with involvement practices, a follow-up review could also focus on mapping service users’ experiences with these practices, provided that gray literature and peer reviewed original research are available to support such a synthesis.

## Conclusions

 This scoping review has shown that regulatory organizations consider service user involvement to be an important aspect of their work. They are experimenting with diverse involvement methods, resulting in increased empirical research over the past decade. Care regulators engage service users for various reasons, employing both reactive and proactive approaches. However, involving service users remains a challenging task, for it necessitates significant time investments and the development of a new skillset for inspectors. Inspectors also express concerns about the subjectivity and representativeness of service user input, which, as research indicates, is downplayed in practice.

 If regulatory efforts are to be aligned with people’s needs and their experiences of health and care services, rather than how providers want to deliver them,^1^ these challenges must be addressed. We hope that the findings from this scoping review emphasize to regulatory organizations and policy-makers more generally that service user involvement should not—and cannot—be treated as a mere tick-box exercise. Creating a rigid typology of “what works when” in terms of service user involvement may be of limited value. Instead, regulators should remain focused on their specific objective when gathering input form service users and/or engaging them in other ways. Regulatory approaches should be tailored accordingly— and, when relevant, aligned with agreed-upon quality standards—to ensure that involvement is both meaningful and impactful. To truly honor the needs and experiential knowledge of service users, regulatory practices could benefit from adopting a more reflexive, less standardized top-down approach.^54^ Prioritizing reciprocal dialogue over adherence to fixed quality standards, this approach entails a collaborative process where service users, inspectors, and other stakeholders collectively identify points of interest and action. Such a process can facilitate mutual learning and understanding among all parties and help forward person-centred service delivery.

## Acknowledgements

 We kindly thank the Netherlands Organization for Scientific Research (NWO) for supporting this study.

## Ethical issues

 Not applicable.

## Conflicts of interest

 In light of full transparency, we deem it important to mention that authors FP and AMP are affiliated with the Dutch HYCI, and JK and HvdB engage in diverse research projects at the HYCI. Several of the empirical studies eligible for selection were carried out within the HYCI. It is crucial to note, however, that the selection process was not influenced by author affiliations with the HYCI, as explicitly outlined in the methods section of the manuscript.

## Endnotes


^[1]^ Text taken from Care Quality Commission’s (CQC) strategy report, 2022. The CQC is the regulator of health and social care services in England.

## Supplementary files


Supplementary file 1. Search Strategies Per Database.
