# Supplementary material for: Involving Service Users in Care Regulation: A Scoping Review of Empirical Literature
Source: Int J Health Policy Manag. 2025 Mar 18;14:8509. doi: 10.34172/ijhpm.8509 (PMC12089843; doi:10.34172/ijhpm.8509)
Supplement: Supplementary file 1 — Search Strategies Per Database. [file ijhpm-14-8509-s001.pdf]

Article title: Involving Service Users in Care Regulation: A Scoping Review of Empirical Literature

Journal name: International Journal of Health Policy and Management (IJHPM)

Authors' information: Josje Kok[1]*, Flora Palimetaki[2], Nada Akrouh[1], Linda Schoonmade[3], Hester van de Bovenkamp[1], Anne Margriet Pot[1,2,4]

[1]Erasmus School of Health Policy & Management, Erasmus University Rotterdam, Rotterdam, The Netherlands.

[2]Dutch Health and Youth Care Inspectorate, Utrecht, The Netherlands.

[3]University Library Vrije Universiteit, Amsterdam, The Netherlands.

[4]North-West University, Vanderbijlpark, South-Africa.

***Correspondence to:** Josje Kok; Email: kok@eshpm.eur.nl

Citation: Kok J, Palimetaki F, Akrouh N, Schoonmade L, van de Bovenkamp H, Pot AM. Involving service users in care regulation: a scoping review of empirical literature. Int J Health Policy Manag. 2025;14:8509. doi:10.34172/ijhpm.8509

Supplementary file 1. Search Strategies Per Database

PubMed

#1 Care

"Health Services"[Mesh] OR "Health Facilities"[Mesh] OR "Residential Facilities"[Mesh] OR "Housing for the Elderly"[Mesh] OR "Geriatrics"[Mesh] OR "Geriatric Psychiatry"[Mesh] OR "Social Welfare"[Mesh] OR "healthcare provider*"[tiab] OR "care provider*"[tiab] OR "healthcare worker*"[tiab] OR "healthcare facilit*"[tiab] OR "health facilit*"[tiab] OR "health care facilit*"[tiab] OR "healthcare organization*"[tiab] OR "healthcare organization*"[tiab] OR "health care organization*"[tiab] OR "health care organization*"[tiab] OR "healthcare organisation*"[tiab] OR "health care organisation*"[tiab] OR "health care organisation*"[tiab] OR "health care service*"[tiab] OR "healthcare service*"[tiab] OR "nursing home*"[tiab] OR "elderly care"[tiab] OR "elderly healthcare"[tiab] OR "care for the elder*"[tiab] OR "old age home*"[tiab] OR "home for the aged"[tiab] OR "geriatric care"[tiab] OR "gerontolog*"[tiab] OR "psychogeriatric nursing"[tiab] OR "geriatric nursing"[tiab] OR "hospital*"[tiab] OR "clinic*"[tiab] OR "long-term care"[tiab] OR "care home*"[tiab] OR "home care"[tiab] OR "home healthcare"[tiab] OR "domiciliary care"[tiab] OR "hospice*"[tiab] OR "palliative nursing"[tiab] OR "day care"[tiab] OR "partial hospitali*"[tiab] OR "assisted living facilit*"[tiab] OR "social care"[tiab] OR "social welfare"[tiab] OR "community service*"[tiab] OR "mental health care"[tiab] OR "mental care"[tiab] OR "mental healthcare"[tiab] OR "disabled care"[tiab] OR "care for disabled"[tiab] OR "disability care"[tiab] OR "psychiatric care"[tiab]

#2 Inspection

"inspect*"[tiab] OR "nursing home compare"[tiab] OR "NHC"[tiab] OR "Care Quality Commission*"[tiab] OR "CQC"[tiab] OR "national regulat*"[tiab] OR "state regulat*"[tiab] OR "federal regulat*"[tiab] OR "government regulat*"[tiab] OR "regulatory bod*"[tiab]

#3 Client experience
"Patient Participation"[Mesh] OR "Empowerment"[Mesh] OR "Patient Satisfaction"[Mesh] OR "Personal Narratives as Topic"[Mesh] OR "Personal Narrative" [Publication Type] OR “expert-by-experience”[tiab] OR “experts-by-experience”[tiab] OR rating*[tiab] OR “personal narrative*”[tiab] OR ((Patient*[tiab] OR citizen*[tiab] OR client*[tiab] OR customer*[tiab] OR user*[tiab]) AND (feedback*[tiab] OR involv*[tiab] OR experienc*[tiab] OR participati*[tiab] OR empower*[tiab] OR engag*[tiab] OR satisf*[tiab]))

Embase

#1 Care
'health service'/exp OR 'health care facility'/exp OR 'geriatrics'/exp OR 'social welfare'/exp OR ‘healthcare provider*’:ti,ab,kw OR ‘care provider*’:ti,ab,kw OR ‘health* worker*’:ti,ab,kw OR ‘health* facilit*’:ti,ab,kw OR ‘health care facilit*’:ti,ab,kw OR ‘health* organi?ation*’:ti,ab,kw OR ‘health care organi?ation*’:ti,ab,kw OR ‘health care service*’:ti,ab,kw OR ‘healthcare service*’:ti,ab,kw OR ‘nursing home*’:ti,ab,kw OR ‘elderly care’:ti,ab,kw OR ‘elderly healthcare’:ti,ab,kw OR ‘care for the elder*’:ti,ab,kw OR ‘old age home*’:ti,ab,kw OR ‘home for the aged’:ti,ab,kw OR ‘geriatric care’:ti,ab,kw OR ‘gerontolog*’:ti,ab,kw OR ‘psychogeriatric nursing’:ti,ab,kw OR ‘geriatric nursing’:ti,ab,kw OR ‘hospital*’:ti,ab,kw OR ‘clinic*’:ti,ab,kw OR ‘long-term care’:ti,ab,kw OR ‘care home*’:ti,ab,kw OR ‘home care’:ti,ab,kw OR ‘home healthcare’:ti,ab,kw OR ‘domiciliary care’:ti,ab,kw OR ‘hospice*’:ti,ab,kw OR ‘palliative nursing’:ti,ab,kw OR ‘day care’:ti,ab,kw OR ‘partial hospitali*’:ti,ab,kw OR ‘assisted living facilit*’:ti,ab,kw OR ‘social care’:ti,ab,kw OR ‘social welfare’:ti,ab,kw OR ‘community service*’:ti,ab,kw OR ‘mental health care’:ti,ab,kw OR ‘mental care’:ti,ab,kw OR ‘mental healthcare’:ti,ab,kw OR ‘disabled care’:ti,ab,kw OR ‘care for disabled’:ti,ab,kw OR ‘disability care’:ti,ab,kw OR ‘psychiatric care’:ti,ab,kw

#2 Inspection
'inspector'/exp OR 'inspection'/exp OR ‘inspect*’:ti,ab,kw OR ‘nursing home compare’:ti,ab,kw OR ‘NHC’:ti,ab,kw OR ‘Care Quality Commission*’:ti,ab,kw OR ‘CQC’:ti,ab,kw OR ‘national regulat*’:ti,ab,kw OR ‘state regulat*’:ti,ab,kw OR ‘federal regulat*’:ti,ab,kw OR ‘government regulat*’:ti,ab,kw OR ‘regulatory bod*’:ti,ab,kw

#3 Client experience
'patient participation'/exp OR 'empowerment'/exp OR 'patient satisfaction'/exp OR ‘expert-by-experience’:ti,ab,kw OR ‘experts-by-experience’:ti,ab,kw OR rating*:ti,ab,kw OR ‘personal narrative*’:ti,ab,kw OR ((patient* OR citizen* OR client* OR customer* OR user*) NEAR/3 (feedback* OR involv* OR experienc* OR participati* OR empower* OR engag* OR satisf*)):ti,ab,kw

---

Cinahl

#1 Care
MH ("Health Services+" OR "Health Facilities+" OR "Geriatrics" OR "Gerontologic Nursing" OR "Geriatric Psychiatry" OR "Dental Care for Aged" OR "Social Welfare+" OR "Long Term Care" OR "Day Care" OR "Gerontologic Care" OR "Home Health Care+" OR "Home Nursing" OR "Terminal Care+" OR "Residential Care+" OR "Residential Facilities+" OR "Hospitals+" OR "Health Services for the Aged" OR "Nursing Homes+" OR "Home Nursing" OR "Hospice Care" OR "Mental Health

Services+") OR TI (“health* provider*” OR “care provider*” OR “health* worker*” OR “health* facilit*” OR “health care facilit*” OR “health* organi?ation*” OR “health care organi?ation*” OR “health care service*” OR “healthcare service*” OR “nursing home*” OR “elderly care” OR “elderly healthcare” OR “care for the elder*” OR “old age home*” OR “home for the aged” OR “geriatric care” OR “gerontolog*” OR “psychogeriatric nursing” OR “geriatric nursing” OR “hospital*” OR “clinic*” OR “long-term care” OR “care home*” OR “home care” OR “home healthcare” OR “domiciliary care” OR “hospice*” OR “palliative nursing” OR “day care” OR “partial hospitali*” OR “assisted living facilit*” OR “social care” OR “social welfare” OR “community service*” OR “mental health care” OR “mental care” OR “mental healthcare” OR “disabled care” OR “care for disabled” OR “disability care” OR “psychiatric care”) OR AB (“health* provider*” OR “care provider*” OR “health* worker*” OR “health* facilit*” OR “health care facilit*” OR “health* organi?ation*” OR “health care organi?ation*” OR “health care service*” OR “healthcare service*” OR “nursing home*” OR “elderly care” OR “elderly healthcare” OR “care for the elder*” OR “old age home*” OR “home for the aged” OR “geriatric care” OR “gerontolog*” OR “psychogeriatric nursing” OR “geriatric nursing” OR “hospital*” OR “clinic*” OR “long-term care” OR “care home*” OR “home care” OR “home healthcare” OR “domiciliary care” OR “hospice*” OR “palliative nursing” OR “day care” OR “partial hospitali*” OR “assisted living facilit*” OR “social care” OR “social welfare” OR “community service*” OR “mental health care” OR “mental care” OR “mental healthcare” OR “disabled care” OR “care for disabled” OR “disability care” OR “psychiatric care”)

#2 Inspection
TI (“inspect*” OR “nursing home compare” OR “NHC” OR “Care Quality Commission*” OR “CQC” OR “national regulat*” OR “state regulat*” OR “federal regulat*” OR “government regulat*” OR “regulatory bod*”) OR AB (“inspect*” OR “nursing home compare” OR “NHC” OR “Care Quality Commission*” OR “CQC” OR “national regulat*” OR “state regulat*” OR “federal regulat*” OR “government regulat*” OR “regulatory bod*”)

#3 Client experience
MH ("Consumer Participation" OR "Empowerment" OR "Patient Satisfaction+" OR "Narratives+") OR TI ((“expert-by-experience” OR “experts-by-experience” OR rating* OR “personal narrative*” OR ((patient* OR citizen* OR client* OR customer* OR user*) AND (feedback* OR involv* OR experienc* OR participati* OR empower* OR engag* OR satisf*))) OR AB (“expert-by-experience” OR “experts-by-experience” OR rating* OR “personal narrative*” OR ((patient* OR citizen* OR client* OR customer* OR user*) AND (feedback* OR involv* OR experienc* OR participati* OR empower* OR engag* OR satisf*)))

PsycInfo

#1 Care
DE ("Health Care Services" OR "Intensive Care" OR "Long Term Care" OR "Mental Health Services" OR "Palliative Care" OR "Hospice" OR "Day Care Centers" OR "Treatment Facilities" OR "Clinics" OR "Community Mental Health Centers" OR "Hospitals" OR "Child Guidance Clinics" OR "Psychiatric Clinics" OR "Walk In Clinics" OR "Hospitals" OR "Psychiatric Hospitals" OR "Nursing Homes" OR "Home Care" OR "Geriatrics" OR "Geriatric Psychiatry" OR "Gerontology" OR "Social Services" OR "Adult Day Care" OR "Community Services" OR "Elder Care" OR "Residential Care Institutions" OR "Halfway Houses" " OR "Orphanages") OR TI (“health* provider*” OR “care provider*” OR “health* worker*” OR “health* facilit*” OR “health care facilit*” OR “health* organi?ation*” OR “health care organi?ation*” OR “health care service*” OR “healthcare service*” OR “nursing home*” OR “elderly

care” OR “elderly healthcare” OR “care for the elder*” OR “old age home*” OR “home for the aged” OR “geriatric care” OR “gerontolog*” OR “psychogeriatric nursing” OR “geriatric nursing” OR “hospital*” OR “clinic*” OR “long-term care” OR “care home*” OR “home care” OR “home healthcare” OR “domiciliary care” OR “hospice*” OR “palliative nursing” OR “day care” OR “partial hospitali*” OR “assisted living facilit*” OR “social care” OR “social welfare” OR “community service*” OR “mental health care” OR “mental care” OR “mental healthcare” OR “disabled care” OR “care for disabled” OR “disability care” OR “psychiatric care”) OR AB (“health* provider*” OR “care provider*” OR “health* worker*” OR “health* facilit*” OR “health care facilit*” OR “health* organi?ation*” OR “health care organi?ation*” OR “health care service*” OR “healthcare service*” OR “nursing home*” OR “elderly care” OR “elderly healthcare” OR “care for the elder*” OR “old age home*” OR “home for the aged” OR “geriatric care” OR “gerontolog*” OR “psychogeriatric nursing” OR “geriatric nursing” OR “hospital*” OR “clinic*” OR “long-term care” OR “care home*” OR “home care” OR “home healthcare” OR “domiciliary care” OR “hospice*” OR “palliative nursing” OR “day care” OR “partial hospitali*” OR “assisted living facilit*” OR “social care” OR “social welfare” OR “community service*” OR “mental health care” OR “mental care” OR “mental healthcare” OR “disabled care” OR “care for disabled” OR “disability care” OR “psychiatric care”) OR KW (“health* provider*” OR “care provider*” OR “health* worker*” OR “health* facilit*” OR “health care facilit*” OR “health* organi?ation*” OR “health care organi?ation*” OR “health care service*” OR “healthcare service*” OR “nursing home*” OR “elderly care” OR “elderly healthcare” OR “care for the elder*” OR “old age home*” OR “home for the aged” OR “geriatric care” OR “gerontolog*” OR “psychogeriatric nursing” OR “geriatric nursing” OR “hospital*” OR “clinic*” OR “long-term care” OR “care home*” OR “home care” OR “home healthcare” OR “domiciliary care” OR “hospice*” OR “palliative nursing” OR “day care” OR “partial hospitali*” OR “assisted living facilit*” OR “social care” OR “social welfare” OR “community service*” OR “mental health care” OR “mental care” OR “mental healthcare” OR “disabled care” OR “care for disabled” OR “disability care” OR “psychiatric care”)

#2 Inspection
TI (“inspect*” OR “nursing home compare” OR “NHC” OR “Care Quality Commission*” OR “CQC” OR “national regulat*” OR “state regulat*” OR “federal regulat*” OR “government regulat*” OR “regulatory bod*”) OR AB (“inspect*” OR “nursing home compare” OR “NHC” OR “Care Quality Commission*” OR “CQC” OR “national regulat*” OR “state regulat*” OR “federal regulat*” OR “government regulat*” OR “regulatory bod*”) OR KW (“inspect*” OR “nursing home compare” OR “NHC” OR “Care Quality Commission*” OR “CQC” OR “national regulat*” OR “state regulat*” OR “federal regulat*” OR “government regulat*” OR “regulatory bod*”)

#3 Client experience
DE ("Client Participation" OR "Empowerment" OR "Client Satisfaction" OR "Narratives" OR "Rating") OR TI (“expert-by-experience” OR “experts-by-experience” OR rating* OR “personal narrative*” OR ((patient* OR citizen* OR client* OR customer* OR user*) AND (feedback* OR involv* OR experienc* OR participati* OR empower* OR engag* OR satisf*)) OR AB (“expert-by-experience” OR “experts-by-experience” OR rating* OR “personal narrative*” OR ((patient* OR citizen* OR client* OR customer* OR user*) AND (feedback* OR involv* OR experienc* OR participati* OR empower* OR engag* OR satisf*)) OR KW (“expert-by-experience” OR “experts-by-experience” OR rating* OR “personal narrative*” OR ((patient* OR citizen* OR client* OR customer* OR user*) AND (feedback* OR involv* OR experienc* OR participati* OR empower* OR engag* OR satisf*))

Scopus

#1 Care

TITLE-ABS-KEY (“health* provider*” OR “care provider*” OR “health* worker*” OR “health* facilit*” OR “health care facilit*” OR “health* organi?ation*” OR “health care organi?ation*” OR “health care service*” OR “healthcare service*” OR “nursing home*” OR “elderly care” OR “elderly healthcare” OR “care for the elder*” OR “old age home*” OR “home for the aged” OR “geriatric care” OR “gerontolog*” OR “psychogeriatric nursing” OR “geriatric nursing” OR “hospital*” OR “clinic*” OR “long-term care” OR “care home*” OR “home care” OR “home healthcare” OR “domiciliary care” OR “hospice*” OR “palliative nursing” OR “day care” OR “partial hospitali*” OR “assisted living facilit*” OR “social care” OR “social welfare” OR “community service*” OR “mental health care” OR “mental care” OR “mental healthcare” OR “disabled care” OR “care for disabled” OR “disability care” OR “psychiatric care”)

#2 Inspection
TITLE-ABS-KEY (“inspect*” OR “nursing home compare” OR “NHC” OR “Care Quality Commission*” OR “CQC” OR “national regulat*” OR “state regulat*” OR “federal regulat*” OR “government regulat*” OR “regulatory bod*”)

#3 Client experience
TITLE-ABS-KEY ((“expert-by-experience” OR “experts-by-experience” OR rating* OR “personal narrative*”) OR ((patient* OR citizen* OR client* OR customer* OR user*) W/3 (feedback* OR involv* OR experienc* OR participati* OR empower* OR engag* OR satisf*)))
